# Long-term prognostic value of whole-heart coronary magnetic resonance angiography

**DOI:** 10.1186/s12968-021-00749-w

**Published:** 2021-05-17

**Authors:** Satoshi Nakamura, Masaki Ishida, Kei Nakata, Yasutaka Ichikawa, Shinichi Takase, Masafumi Takafuji, Haruno Ito, Shiro Nakamori, Tairo Kurita, Kaoru Dohi, Hajime Sakuma

**Affiliations:** 1 Department of Radiology, Mie University Hospital, 2-174 Edobashi, Tsu, Mie 514-8507 Japan; 2 Department of Cardiology and Nephrology, Mie University Hospital, Tsu, Mie Japan

**Keywords:** Coronary artery disease, Coronary magnetic resonance angiography, Long-term prognostic value

## Abstract

**Background:**

Coronary magnetic resonance angiography (CMRA) allows non-ionizing visualization of luminal narrowing in coronary artery disease (CAD). Although a prior study showed the usefulness of CMRA for risk stratification in short-term follow-up, the long-term prognostic value of CMRA remains unclear. The purpose of this study was to evaluate the long-term prognostic value of CMRA.

**Methods:**

A total of 506 patients without history of myocardial infarction or prior coronary artery revascularization underwent free-breathing whole-heart CMRA between 2009 and 2015. Images were acquired using a 1.5 T or 3 T scanner and visually evaluated as the consensus decisions of two observers. Obstructive CAD on CMRA was defined as luminal narrowing of ≥ 50% in at least one coronary artery. Major adverse cardiac events (MACE) comprised cardiac death, nonfatal myocardial infarction, and unstable angina.

**Results:**

Obstructive CAD on CMRA was observed in 214 patients (42%). During follow-up (median, 5.6 years), 31 MACE occurred. Kaplan–Meier curve analysis revealed a significant difference in event-free survival between patients with and without obstructive CAD for MACE (log-rank, p = 0.003) and cardiac death (p = 0.012). Annualized event rates for MACE in patients with no obstructive CAD, 1-vessel disease, 2-vessel disease, and left-main or 3-vessel disease were 0.6%, 1.5%, 2.3%, and 3.6%, respectively (log-rank, p = 0.003). Cox proportional hazard regression analysis showed that, among obstructive CAD on CMRA and clinical risk factors (age, sex, hypertension, diabetes, dyslipidemia, smoking, and family history of CAD), obstructive CAD and diabetes were significant predictors of MACE (hazard ratios, 2.9 [p = 0.005] and 2.2 [p = 0.034], respectively). In multivariate analysis, obstructive CAD remained an independent predictor (adjusted hazard ratio, 2.6 [p = 0.010]) after adjusting for diabetes. Addition of obstructive CAD to clinical risk factors significantly increased the global chi-square result from 8.3 to 13.8 (p = 0.022).

**Conclusions:**

In long-term follow-up, free breathing whole heart CMRA allows non-invasive risk stratification for MACE and cardiac death and provides incremental prognostic value over conventional risk factors in patients without a history of myocardial infarction or prior coronary artery revascularization. The presence and severity of obstructive CAD detected by CMRA were associated with worse prognosis. Importantly, patients without obstructive CAD on CMRA displayed favorable prognosis.

**Supplementary Information:**

The online version contains supplementary material available at 10.1186/s12968-021-00749-w.

## Background

Coronary artery disease (CAD) is a leading cause of morbidity and mortality around the world [1]. Early diagnosis of this pathology may be helpful in guiding clinical management. Invasive coronary angiography is currently regarded as the gold standard for detecting CAD, but is costly and is associated with risk of complications. Coronary computed tomography angiography (CCTA) has been widely used for the non-invasive evaluation of coronary atherosclerosis. Despite its utility in the assessment of coronary morphology [2, 3] and risk stratification for future cardiac events [4, 5], CCTA necessitates radiation exposure. Coronary magnetic resonance angiography (CMRA) has been developed for > 25 years as a non-invasive, non-ionizing alternative for visualizing the coronary artery lumen, showing steady improvement with promising results in the diagnosis of narrowing in the coronary arteries [6–9]. However, data on clinical outcomes among patients who have undergone CMRA are limited. Although Yoon et al. demonstrated the utility of whole-heart CMRA for risk stratification during follow-up (median, 25 months) [10], the long-term prognostic value of CMRA remains unclear. The aim of this study was thus to evaluate the long-term prognostic value of free-breathing whole-heart CMRA.

## Methods

### Study population

**Fig. 1:**
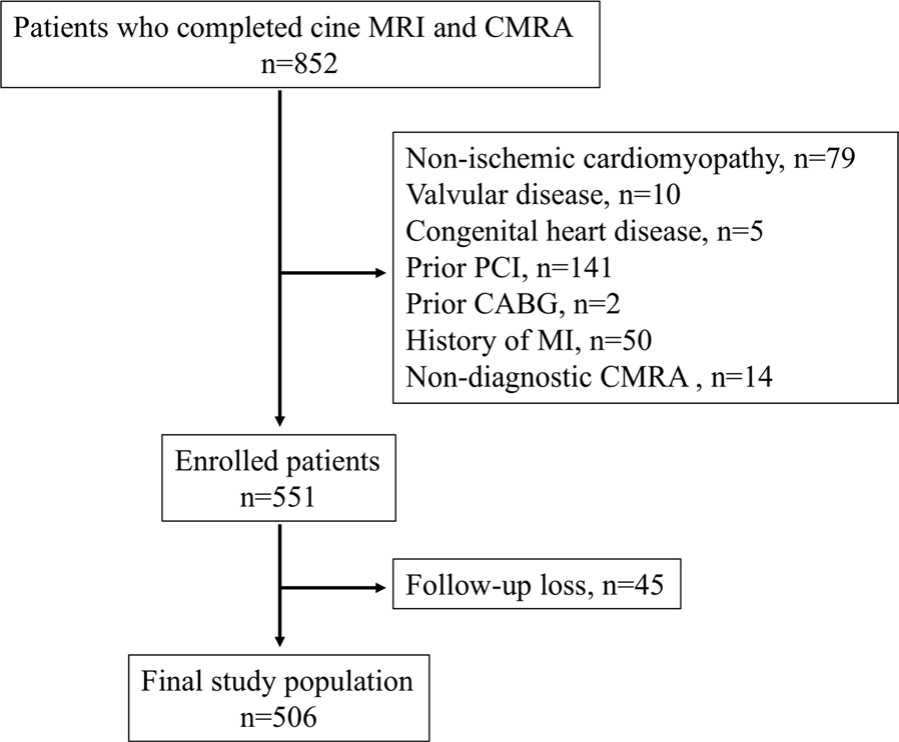
Flow chart of patient selection. This figure shows a flow chart of the patient selection in this study. *CABG*  coronary artery bypass grafting, *CMRA*  coronary magnetic resonance angiography, *MI*  myocardial infarction, *PCI* percutaneous coronary intervention

This retrospective study included 882 patients ≥ 45 years old who were referred for whole-heart CMRA for the indications shown in Additional file 1: Table S1 between January 2009 and February 2015 at our hospital. Since there were 30 unsuccessful cases (3.4%), 852 patients completed a CMRA. Of those 852 patients, we excluded 301 patients with non-ischemic cardiomyopathy (n = 79), valvular disease (n = 10), congenital heart disease (n = 5), previous coronary artery revascularization via percutaneous coronary intervention (n = 141) or coronary artery bypass grafting (n = 2), history of myocardial infarction (MI) (n = 50), and non-diagnostic CMRA (n = 14) (Fig. 1). Therefore, 551 patients underwent follow-up after CMRA. The pre-test likelihood of CAD was determined using the Diamond and Forrester method, as previously described [11]. The institutional review board in our hospital approved the protocols for this retrospective study and waived the need to obtain individual consent based on the retrospective design (reference number: H2019-184).

### Image acquisition

All CMRA were performed using a 1.5 T (n = 205) or 3 T (n = 301) scanner (Achieva 1.5 T/Achieva 3.0 T; Philips Healthcare, Best, the Netherlands). Radiofrequency reception was performed using a 32-element cardiac coil. CMRA was performed as a part of a: (1) cine CMR and CMRA protocol (n = 23); (2) cine CMR, late gadolinium-enhanced (LGE), and CMRA protocol (n = 25); or (3) cine CMR, stress perfusion, LGE, and CMRA protocol (n = 458) using a 1.5 T or 3 T scanner. The protocol (1), performed by the 1.5 T CMR scanner alone, used no contrast agent. The protocols (2) and (3) were performed with gadolinium-based contrast agent using 1.5 T or 3 T scanners. To assess left ventricular (LV) volume, function, and mass, breath-holding cine CMR was performed with a segmented balanced steady-state free precession (bSSFP) sequence in the short-axis planes covering the entire LV (slice thickness, 10 mm; cardiac phases, 20; repetition time (TR), 3.2 ms; echo time (TE), 1.6 ms; flip angle (FA), 55°; field-of-view (FOV), 35 × 35 cm; acquisition matrix, 192 × 192; reconstruction matrix, 256 × 256 for 1.5 T scanner; TR, 2.8 ms; TE, 1.4 ms; FA, 55°; FOV, 35 × 35 cm; acquisition matrix size, 176 × 308; reconstruction matrix, 352 × 352 for 3 T scanner). Acquisition methods for stress perfusion CMR and LGE are presented in the Additional file 1: Supplemental Methods.

Sublingual isosorbide denitrate 5 mg was administered to all subjects before CMRA acquisition. Beta-blockers were not used. CMRA was acquired after cine CMR at the protocol (1) or after LGE imaging (2) and (3). To monitor motion of the right coronary artery (RCA), transaxial cine CMR was performed under free breathing for 50 cardiac phases. A patient-specific acquisition window in the cardiac cycle was set during either systole or diastole, depending on the phase of minimal motion of the RCA [8]. Free-breathing, navigator-gated 3-dimensional (3D) whole-heart CMRA was obtained with a segmented bSSFP sequence using T2 preparation, fat saturation, and radial k-space sampling at 1.5 T (TR, 4.1 ms; TE, 2.4 ms; FA, 80°; full Fourier encoding; excitations per cardiac cycle, 8–12; FOV, 280 × 280 mm; acquisition matrices, 224 × 174; reconstruction matrices, 512 × 512; acquisition slice thickness, 1.7 mm; reconstruction slice thickness, 0.85 mm; SENSE factor, 4) or with a turbo field echo (TFE) sequence using T2 preparation, fat saturation, and radial k-space sampling at 3 T (TR, 3.8 ms; TE, 1.7 ms; FA, 15°; full Fourier encoding; excitations per cardiac cycle, 9–14; FOV, 330 × 280 mm; acquisition matrices, 256 × 193; reconstruction matrices, 512 × 512; acquisition slice thickness, 1.6 mm; reconstruction slice thickness, 0.8 mm; SENSE factor, 3.3). Slab thickness was adapted for each patient to cover the entire heart. The navigator gating window was ± 2.5 mm.

### Image analysis

CMR images were analyzed by two independent, blinded observers using cvi42 software (Circle Cardiovascular Imaging, Calgary, Alberta, Canada). At end-diastole and end-systole, endocardial LV borders were manually traced in contiguous images from short-axis cine CMR covering from apex to mitral valve planes to calculate LV end-diastolic volume (LVEDV) and end-systolic volume (LVESV) and ejection fraction (LVEF). After tracing epicardial LV borders at end-diastole, LV mass was calculated as the sum of myocardial volume at end-diastole multiplied by the specific gravity (1.05 g/mL) of myocardial tissue.

**Fig. 2:**
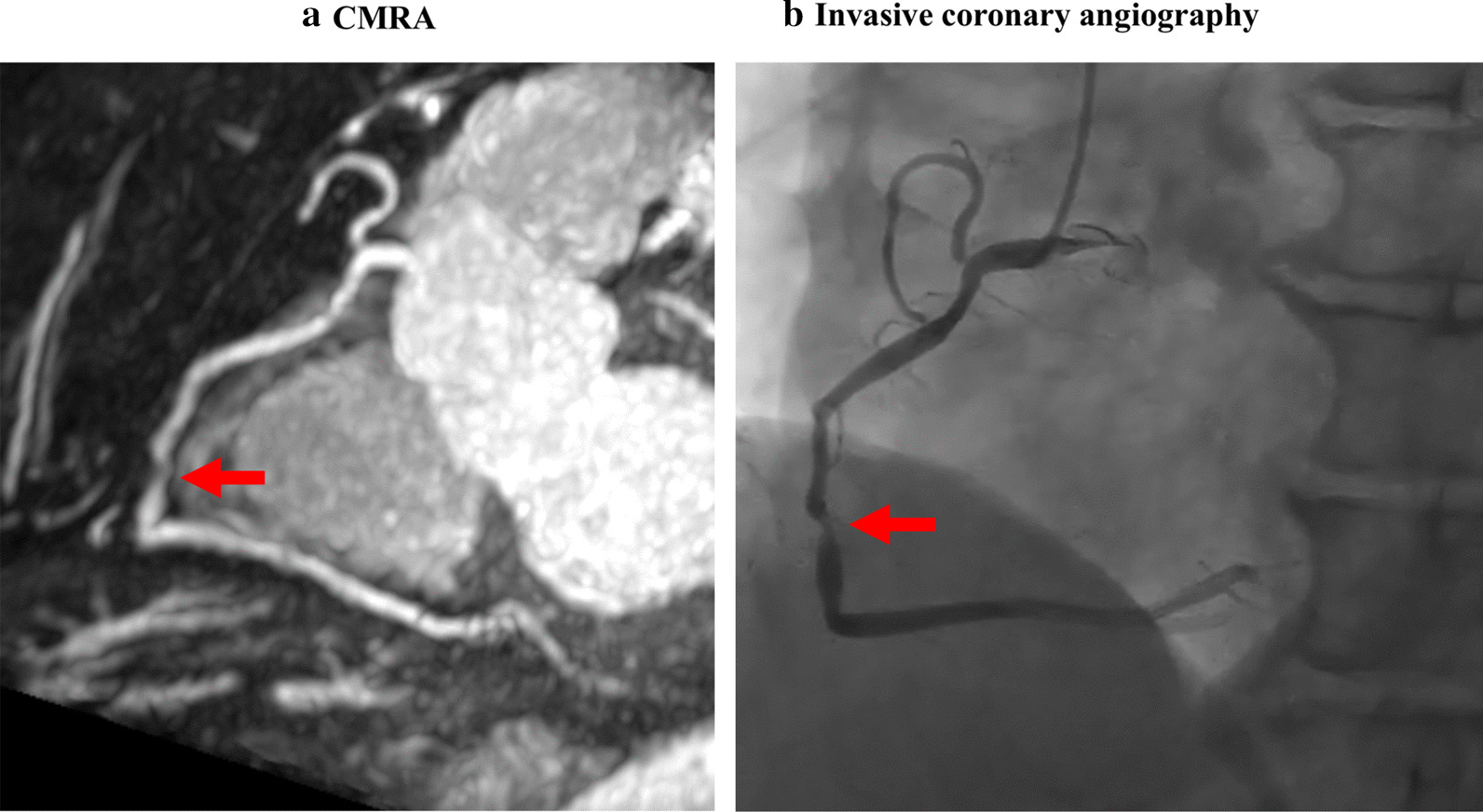
A case in a 66-year-old female with multiple risk factors. The figures illustrate **a** thin-slab maximum intensity projection image and **b** invasive coronary angiography that show significant stenosis (red arrow) in the mid portion of the right coronary artery

Two observers blinded to clinical information evaluated the coronary arteries with a diameter ≥ 2 mm on whole-heart CMRA using sliding thin-slab maximum intensity projection. All coronary arteries were included for the evaluation regardless the image quality of coronary MRA. The presence or absence of significant luminal narrowing (≥ 50% diameter narrowing) was interpreted using an intention-to-read approach [7, 10, 12]. Disagreements between the two observers were settled by consensus readings. Figure 2 shows the representative example of a patient with obstructive CAD.

### Follow-up

Follow-up information was collected through a review of hospital records or telephone interviews blindly to CMRA results. Major adverse cardiac events (MACE) comprised cardiac death, non-fatal MI, and unstable angina [13–15]. Cardiac death was defined as death caused by acute MI, ventricular arrhythmias, or heart failure. Nonfatal MI was defined as prolonged angina accompanied by new electrocardiogram (ECG) abnormalities and increased cardiac biomarkers. Unstable angina was defined as new-onset, worsening, or rest angina requiring hospital admission.

### Statistical analysis

Continuous variables are presented as mean ± standard deviation and categorical variables are expressed as frequency and percentage. The influence of predictors on MACE was determined using Cox proportional hazards regression analysis, with the results reported as hazard ratios (HRs) with 95% confidence intervals (CIs). Univariate Cox proportional hazards regression analysis was performed to identify potential predictors of MACE. Multivariate Cox proportional hazards regression analysis was performed using the “enter” method for variables showing values of p < 0.05 in univariate analysis to determine independent predictors of MACE. The incremental value of obstructive CAD on CMRA over clinical risk factors was evaluated by using the global chi-square test. Kaplan–Meier curves were used to estimate event-free rates for MACE and cardiac death. Differences between time-to-event curves were compared using the log-rank test. Annualized event rates were calculated by dividing 5-year Kaplan–Meier event rates by 5. Two-sided values of p < 0.05 were considered significant. All analyses were performed using the SPSS (version 23.0, Statistical Package for the Social Sciences, International Business Machines, Inc., Armonk, New York, USA).

## Results

Of the 551 patients, 45 (8.2%) were lost to follow-up. The final study population thus comprised 506 patients (67 ± 9 years, 56% men) Body mass index, coronary risk factors, pre-test likelihood of CAD, and the number of early revascularizations were shown in Table 1. Obstructive CAD on CMRA was observed in 214 patients (42%). One-vessel, two-vessel, and three-vessel disease were found in 118 (23%), 67 (13%), and 29 (6%) patients, respectively. Mean scan time for CMRA was 11.9 ± 4.0 min. Results by CMR imaging are presented in Table 2. During follow-up (median, 5.6 years), MACE was observed in 31 patients (cardiac death, n = 12; non-fatal MI, n = 1; unstable angina, n = 18). Non-cardiac death was observed in 28 patients.

**Table 1:** Patient characteristics

Characteristic	All patients (n = 506)
Male	281 (56)
Age (mean ± SD)	67 ± 9
Body mass index (mean ± SD)	23 ± 3.7
*Coronary risk factors*	
Hypertension	320 (63)
Dyslipidemia	266 (53)
Diabetes	149 (29)
Smoking	198 (39)
Family history of CAD	68 (13)
*Pre-test likelihood of CAD*	
Low	228 (45)
Intermediate	182 (36)
High	96 (19)
Early (< 90 days) revascularization	44 (9)

Except where indicated, data are numbers of patients (percentages)

*CAD* coronary artery disease

**Table 2:** Imaging results

Parameters	All patients (n = 506)
Heart rate, beats/min	68 ± 12
LVEDV index, ml/m^2^	78 ± 20
LVESV index, ml/m^2^	32 ± 16
LVEF (%)	61 ± 9
LV mass index, g/m^2^	55 ± 16
Obstructive CAD	214 (42)
One-vessel disease	118 (23)
Two-vessel disease	67 (13)
Three-vessel disease	29 (6)
Scan time of CMRA (min)	11.9 ± 4.0

Except where indicated, data are numbers of patients (percentages)

*CAD*  coronary artery disease, *CMRA*  coronary magnetic resonance angiography, *LVEDV* left ventricular end-diastolic volume, *LVEF*  left ventricular ejection fraction, *LVESV*  left ventricular end-systolic volume

**Fig. 3:**
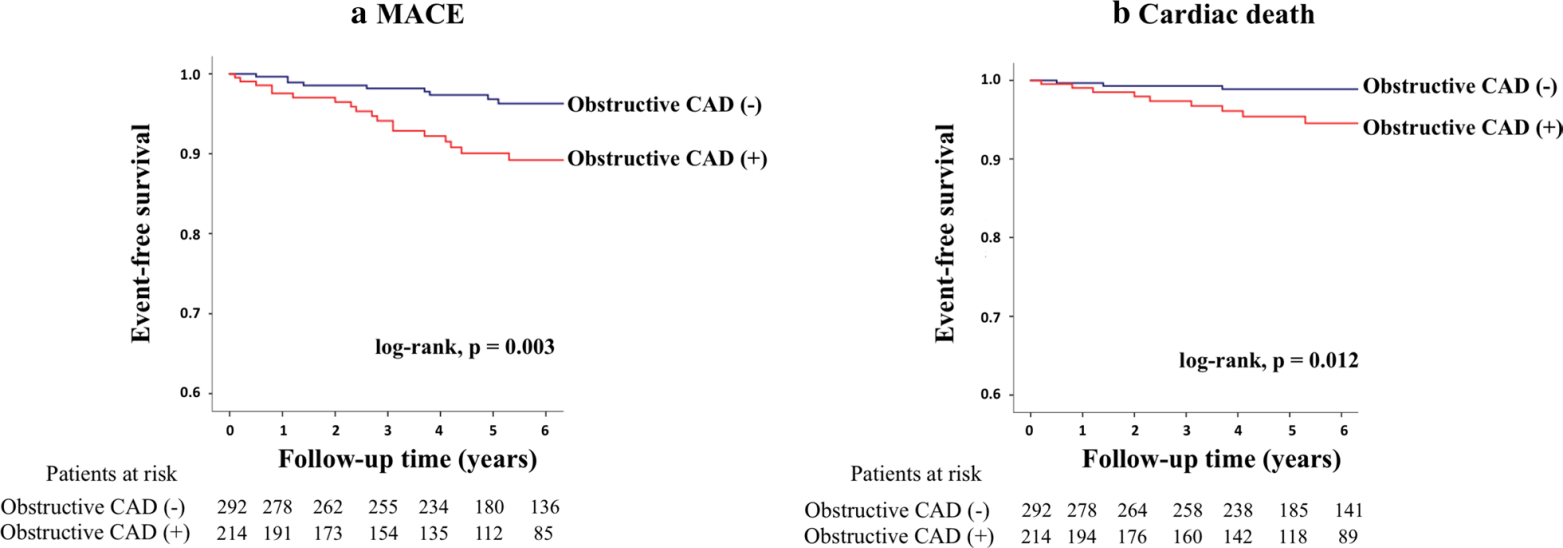
Long-term risk stratification by CMRA. The figures show event-free survival curves in patients stratified by presence or absence of obstructive CAD on CMRA for **a** MACE and **b** cardiac death. *CAD* coronary artery disease, *CMRA* coronary magnetic resonance angiography, *MACE*  major cardiac adverse events

**Fig. 4:**
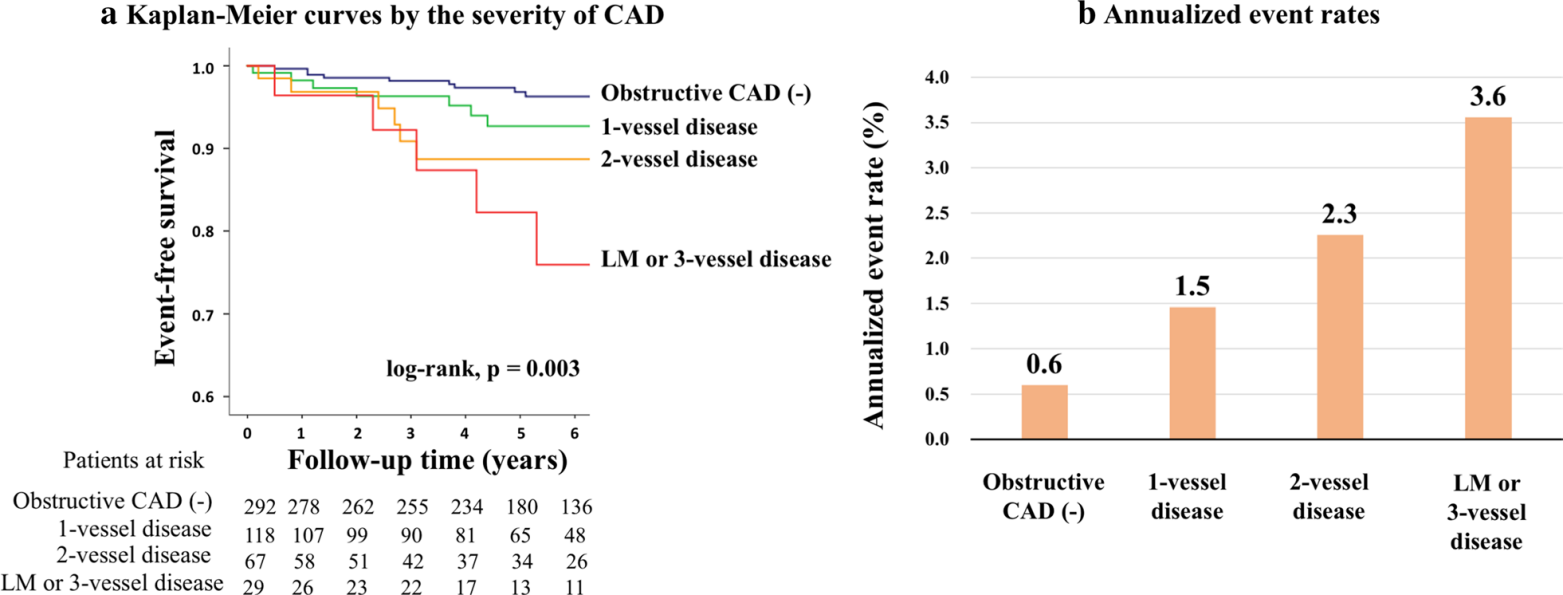
Risk stratification by the severity of CAD. The figures show **a** event-free survival curves in patients stratified by the severity of CAD on CMRA for MACE and **b** annualized event rates according to the severity of CAD. *CAD*  coronary artery disease, *CMRA* coronary magnetic resonance angiography, *LM* left main, *MACE*  major cardiac adverse events

Kaplan–Meier curve analysis revealed a significant difference in event-free survival between patients with and without obstructive CAD on CMRA for MACE (log-rank, p = 0.003; Fig. 3a) and cardiac death (p = 0.012; Fig. 3b). Annualized event rates in patients with and without obstructive CAD on CMRA were 2.0% and 0.6%, respectively, for MACE, and 0.9% and 0.4%, respectively, for cardiac death. Figure 4a shows risk stratification by severity of CAD (log-rank, p = 0.003). Annualized event rates for MACE in patients with 1-vessel disease, 2-vessel disease, and left-main or 3-vessel disease were 1.5%, 2.3%, and 3.6%, respectively (Fig. 4b).

**Fig. 5:**
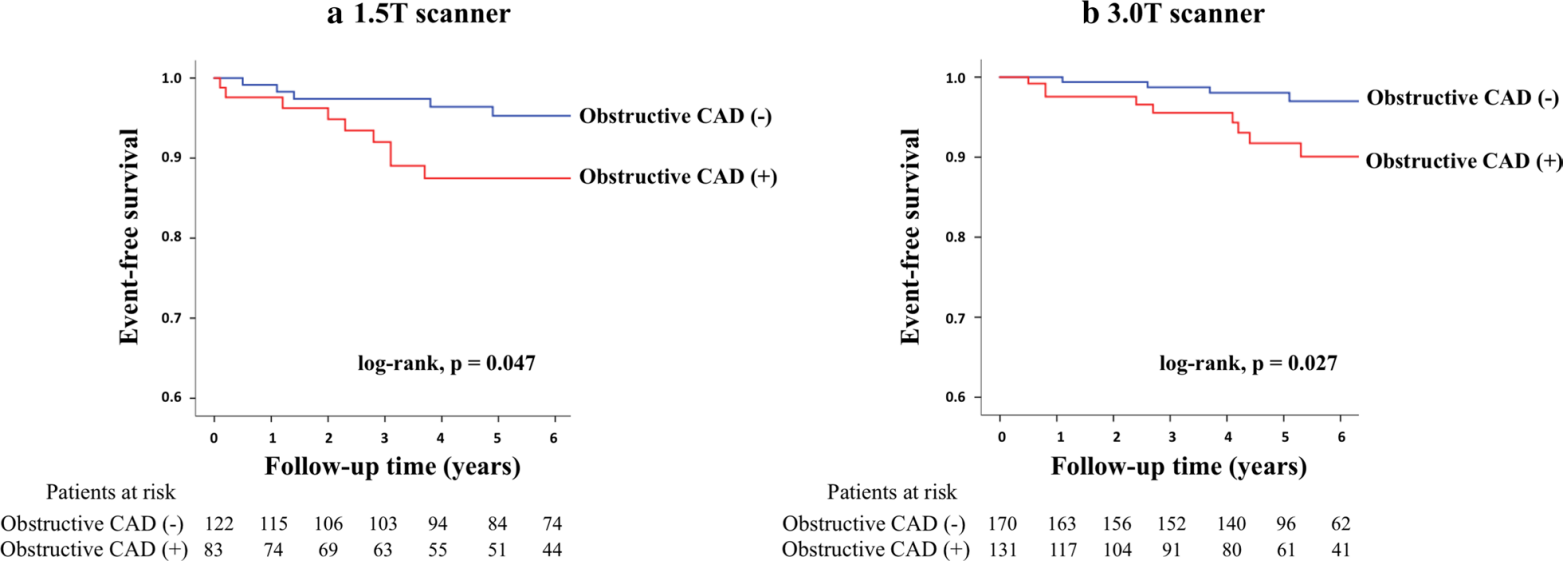
Event-free survival analysis according to 1.5 T or 3 T scanners. The figures show event-free survival curves for presence or absence of obstructive CAD among patients who underwent an CMR scan by field strength. **a** 1.5 T or **b** 3 T scanners. *CAD*  coronary artery disease

Event-free survival analysis according to 1.5 T or 3 T CMRA was shown in Fig. 5. Among patients who underwent an CMR using a 1.5 T or 3 T scanner, there was a significant difference (log-rank; p = 0.047 or 0.027, respectively) in event-free survival for MACE between those with and without obstructive CAD on CMRA.

Results for univariate Cox proportional hazard regression analysis for predicting MACE are listed in Table 3. Age, sex, LVEF < 50%, LVEDV index exceeding the median (> 76 ml/m2), and coronary artery disease risk factors excluding diabetes showed no significant results. Obstructive CAD on CMRA and diabetes were significant predictors of MACE (HR, 2.9; 95%CI, 1.4–6.1; p = 0.005 and HR, 2.2; 95%CI, 1.1–4.4; p = 0.034, respectively). Multivariate analysis showed that obstructive CAD on CMRA remained an independent predictor (adjusted HR, 2.6; 95%CI, 1.3–5.6; p = 0.010) after adjusting for diabetes (adjusted HR, 1.9; 95%CI, 0.9–3.8; p = 0.089).

**Table 3:** Cox proportional hazard regression analysis

Predictor	Univariate		Multivariate	
	HR (95% CI)	p value	HR (95% CI)	p value
Sex	1.1 (0.6–2.3)	0.705		
Age	1.0 (0.9–1.1)	0.609		
Hypertension	1.2 (0.6–2.6)	0.620		
Dyslipidemia	1.9 (0.9–4.0)	0.100		
Diabetes	2.2 (1.1–4.4)	0.034	1.9 (0.9–3.8)	0.089
Smoking	1.2 (0.6–2.4)	0.695		
Family history of CAD	2.0 (0.9–4.6)	0.113		
LVEF < 50%	1.4 (0.3–6.7)	0.636		
LVEDV index > median	0.6 (0.2–1.7)	0.338		
Obstructive CAD	2.9 (1.4–6.1)	0.005	2.6 (1.3–5.6)	0.010

*HR* hazard ratio, *CI* confidence interval, *CAD*  coronary artery disease, *CMRA*  coronary magnetic resonance angiography, *LVEDV* left ventricular end-diastolic volume, *LVEF*  left ventricular ejection fraction, *LVESV*  left ventricular end-systolic volume

**Fig. 6:**
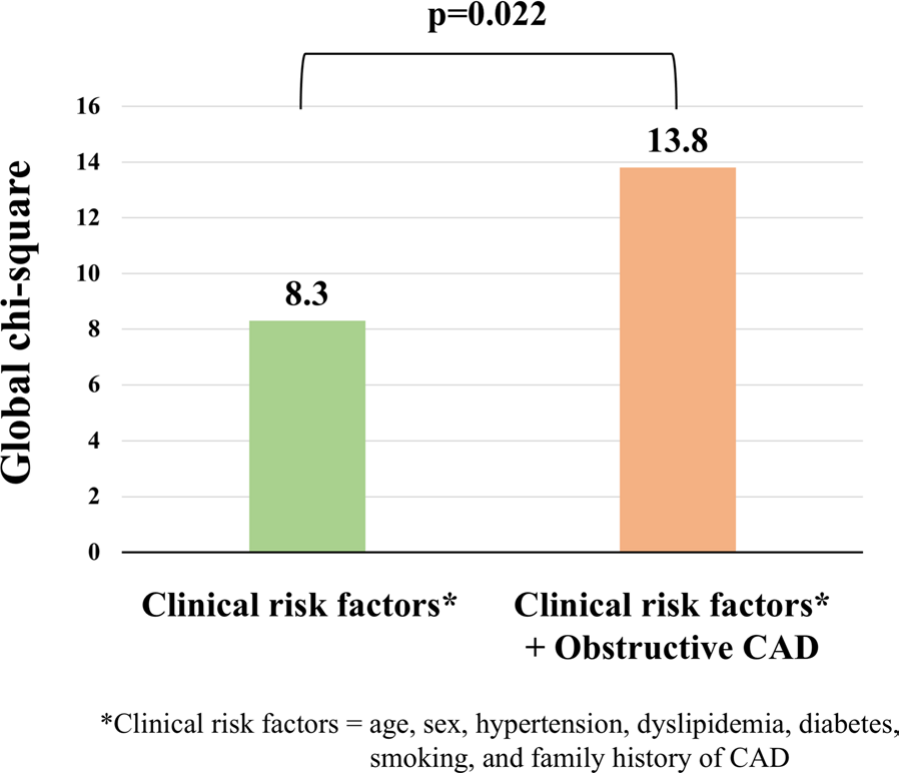
Incremental value of CMRA over clinical risk factors. The figure shows a global chi-square test that demonstrates incremental prognostic value of CMRA over clinical risk factors (age, sex, hypertension, dyslipidemia, diabetes, smoking, and family history of CAD). *CAD*  coronary artery disease, *CMRA* coronary magnetic resonance angiography, *LM* left main, *MACE*  major cardiac adverse events

According to the global chi-square test, addition of obstructive CAD on CMRA to clinical risk factors (age, sex, hypertension, diabetes, dyslipidemia, smoking, and family history of CAD) significantly increased the global chi-square from 8.3 to 13.8 (p = 0.022; Fig. 6).

## Discussion

To the best of our knowledge, this is the first study to evaluate the long-term prognostic value of whole-heart CMRA. During the median follow-up of 5.6 years, demonstration of coronary artery luminal narrowing by CMRA was an independent predictor of MACE. Patients without obstructive CAD on CMRA were at low risk of future cardiac events.

CMRA allows non-invasive assessment of CAD with an acceptable diagnostic accuracy for detecting obstructive CAD [6–9]. A multi-center trial by Kato et al. [7] showed that, in 127 patients with suspected CAD, whole-heart CMRA at 1.5 T allowed non-invasive detection of stenosis ≥ 50% on ICA with high sensitivity (88%) and moderate specificity (72%). Importantly, the negative predictive value of 88% implied that whole-heart CMRA can be used to exclude CAD. A single-center study using 3 T imaging [9] demonstrated that, among 62 patients with suspected CAD, whole-heart CMRA correctly identified patients showing significant stenosis with 92% sensitivity and 83% specificity.

CCTA remains the most common non-invasive clinical technique to noninvasively visualize the coronary arteries. Although CMRA is limited in terms of the lower spatial resolution and longer imaging time, several advantages over CCTA are provided [16]. CMRA does not expose patients to ionizing radiation and can visualize the lumen of the coronary arteries with heavy calcification [17]. The use of a patient-specific acquisition window in the cardiac cycle can provide adequate temporal resolution for each patient, allowing the acquisition of CMRA images without the administration of beta-blockers, even in patients with a high heart rate (> 70 beats/min) [7]. There have been limited data on comparison of the prognostic values of CMRA and CCTA. Hamdan et al. compared CMRA and CCTA in the prognostic value in patients with suspected or known CAD scheduled for invasive coronary angiography [18] and showed that the hazard ratio was 4.69 (95%CI, 1.80–12.24, p = 0.002) for positive versus negative CCTA and 3.17 (95%CI, 1.36–7.36, p = 0.007) for positive versus negative CMRA. Importantly, the absence of coronary stenosis in CMRA or CCTA was associated with low risk for cardiac events.

Yoon et al. showed that, during a median follow-up of 25 months, the presence of significant stenosis (stenosis ≥ 50%) on whole-heart CMRA was strongly associated with future major cardiac events in patients (n = 207) with suspected CAD [10]. Patients with significant stenosis on CMRA exhibited worse prognosis (annualized event rate, 3.9% for severe events; 6.3% for all cardiac events) than patients without significant stenosis (0% for severe events; 0.3% for all cardiac events). Despite those promising results, the study by Yoon et al. was limited in a relatively short follow-up. Since the progression of CAD is gradual, a short-term follow-up is not sufficient to assess prognosis for CAD patients. In fact, there have been several important studies that investigated the long-term prognostic value of CAD-related predictors by CCTA, stress CMR and single photon emission tomography (SPECT) [19, 20]**.** The current study demonstrated the utility of CMRA for risk stratification during a 5-year follow-up in a substantially larger number of subjects. Some MACEs occurred after 2-years follow-up, which implied the importance of long-term duration in this cohort study. In addition, as shown with CCTA [4, 13], this study showed that the severity of CAD has an impact on outcomes, revealing that patients with left-main or 3-vessel disease are at a high risk of MACE.

### Clinical implications

Our results implied that free breathing whole-heart CMRA allowed long-term risk stratification of patients through visualization of coronary arteries without radiation exposure. The presence and severity of obstructive CAD detected using CMRA were associated with worse prognosis. More importantly, patients without obstructive CAD on CMRA displayed an event rate < 1%, consistent with results from studies on the prognostic value of CCTA [4, 21, 22]. In addition, the results of the current study demonstrated additional prognostic value when compared to conventional risk factors. The value of non-invasive imaging in risk stratification remains a hot topic, with debate about whether non-invasive imaging methods should be included in conventional risk stratification tools and guidelines. This study may give a new perspective to that debate by proposing a risk stratification scheme that combines CMRA and conventional risk factors.

### Limitations

Several limitations should be noted in this study. First, this was a single-center, retrospective study. A large, multi-center, prospective study is needed to confirm the present results. Second, we used both 1.5 T and 3 T CMR scanners, which differed in several respects, including diagnostic performance [23]. However, our results showed that CMRA with both 1.5 T and 3 T scanners provided a prognostic value. Third, the present study was performed in patients with an intermediate CAD prevalence of 42%. The findings from this study thus may not be directly extrapolatable to populations with a lower prevalence of CAD. Fourth, since bright-blood CMRA sequences were employed in this study, plaque characteristics were not investigated. Fifth, patients aged under 45 from the study population were excluded, because they often have reasons other than CAD (congenital heart disease, cardiomyopathy, etc.) for undergoing CMR. However, such younger patients have a lot to gain from CMR, which need no radiation exposure, and a further study will be needed to evaluate prognostic value of CMRA in those patients. Sixth, CMRA methods used in the present study did not include newer techniques with higher spatial resolution, 100% efficient acquisitions acquired or reconstructed at isotropic resolution [24–26]. However, since traditional whole-heart CMRA sequences were able to provide such prognostic benefit as shown in the current study, CMRA using more recent technologies is highly promising. Seventh, although CMRA had an acceptable diagnostic accuracy for detection of significant stenosis, CMRA findings were not validated using invasive coronary angiography or compared with CCTA. Eighth, the diagnosis of having obstructive CAD on CMRA may have impact on treatment strategy, especially early revascularization. In the current study, we evaluated a long-term prognostic value of CMRA for predicting "harder" events, not including revascularization. Additionally, the number of early revascularizations in this study was relatively small (9%). Therefore, the impact of revascularization subsequent to CMRA is likely to be limited.

## Conclusions

In a long-term follow-up, free breathing whole-heart CMRA allows non-invasive risk stratification for MACE and cardiac death and provides incremental prognostic value over conventional risk factors. Importantly, patients without obstructive CAD on CMRA displayed favorable prognosis.

## Supplementary Information

**Additional file 1: Table S1.** Indications for CMR.

## Data Availability

Not applicable.

## Abbreviations

bSSFP: Balanced steady-state-free precession
CAD: Coronary artery disease
CCTA: Coronary computed tomography angiography
CI: Confidence interval
CMRA: Coronary magnetic resonance angiography
ECG: Electrocardiogram
HR: Hazard ratio
LGE: Late gadolinium enhancement
LM: Left main coronary artery
LV: Left ventricle/left ventricular
LVEDV: Left ventricular end-diastolic volume
LVEF: Left ventricular ejection fraction
LVESV: Left ventricular end-systolic volume
MACE: Major adverse cardiac event
MI: Myocardial infarction
LV: Left ventricle/left ventricular
RCA: Right coronary artery
TR: Repetition time
TE: Echo time
TFE: Turbo field echo
FA: Flip angle
FOV: Field of view
