# Supplementary material for: Long-term prognostic value of whole-heart coronary magnetic resonance angiography
Source: J Cardiovasc Magn Reson. 2021 May 17;23:56. doi: 10.1186/s12968-021-00749-w (PMC8127259; doi:10.1186/s12968-021-00749-w)
Supplement: Supplementary file 1 — Additional file 1: Table S1. Indications for CMR. [file 12968_2021_749_MOESM1_ESM.docx]

**Table S1. Indications for cardiac MRI**

| **Indications** | **Patients**  **(n = 852)** |
| --- | --- |
| Chest pain | 426 (50) |
| Dyspnea | 60 (7) |
| Multiple risk factors* | 135 (16) |
| Abnormal ECG | 34 (4) |
| LV hypertrophy | 41 (5) |
| LV dysfunction | 52 (6) |
| Abnormal stress test | 5 (1) |
| Follow up of known CAD | 99 (12) |

CAD = coronary artery disease; ECG = electrocardiogram; LV = left ventricular; MRI = magnetic resonance imaging

*Multiple risk factors were defined as having two or more of the risk factors including hypertension, diabetes, dyslipidemia, smoking, and family history of CAD.

**Supplemental Methods**

Stress perfusion MRI consists of 3 or 4 short-axis slices acquired every cardiac cycle or every second cardiac cycles with a saturation-recovery balanced SSFP or turbo field echo (TFE) sequence for a 1.5- or 3.0-T scanner (TR, 3.0/2.9 ms; TE, 1.5/1.4 ms; FA, 40/20°; FOV, 36 × 32/32 × 29 cm; acquisition matrix, 192 × 153/224 × 131; SENSE factor, 2.8/2.5; time between saturation preparation pulse and center of k-space acquisition, 200/110 ms; duration of image data acquisition, 211/141 ms; slice thickness section, 8/10 mm), respectively. For stress perfusion MRI, dynamic MRI was initiated 3 min after starting ATP infusion (0.16 mg/kg/min) and continued for 1 min. Gadoterate meglumine (Gd-DOTA; Guerbet Japan, Tokyo, Japan) was injected at a dose of 0.03 mmol/kg and a flow rate of 4 mL/s, followed by a 20-mL saline flush for stress perfusion MRI. Rest perfusion MRI was performed with an identical set-up at 10 min after stress perfusion MRI. LGE MRI was performed in the same LV short-axis planes as cine MRI using a 3D inversion recovery TFE sequence 5–10 min after intravenous administration of Gd-DOTA with the cumulative dose of 0.15 mmol/kg for a 1.5- or 3-T scanner (TR, 3.8/4.6 ms; TE, 1.2/2.2 ms; FA, 15°/15°; FOV, 40×36×5/38×34×5 cm; acquisition matrix, 224×156×5/240×192×5; reconstructed matrix, 256×256×10/384×384×10; SENSE factor, 2/3; TFE factor, 24/33, respectively).
